# Improve the Hunger Games search algorithm to optimize the GoogleNet model

**DOI:** 10.1371/journal.pone.0305653

**Published:** 2024-08-16

**Authors:** Yanqiu Li, Shizheng Qu, Huan Liu

**Affiliations:** 1 School of Data Science and Artificial Intelligence, Jilin Engineering Normal University, Changchun, China; 2 College of Information Technology, Jilin Agricultural University, Changchun, China; New York University Abu Dhabi, UNITED ARAB EMIRATES

## Abstract

The setting of parameter values will directly affect the performance of the neural network, and the manual parameter tuning speed is slow, and it is difficult to find the optimal combination of parameters. Based on this, this paper applies the improved Hunger Games search algorithm to find the optimal value of neural network parameters adaptively, and proposes an ATHGS-GoogleNet model. Firstly, adaptive weights and chaos mapping were integrated into the hunger search algorithm to construct a new algorithm, ATHGS. Secondly, the improved ATHGS algorithm was used to optimize the parameters of GoogleNet to construct a new model, ATHGS-GoogleNet. Finally, in order to verify the effectiveness of the proposed algorithm ATHGS and the model ATHGS-GoogleNet, a comparative experiment was set up. Experimental results show that the proposed algorithm ATHGS shows the best optimization performance in the three engineering experimental designs, and the accuracy of the proposed model ATHGS-GoogleNet reaches 98.1%, the sensitivity reaches 100%, and the precision reaches 99.5%.

## Introduction

The advantages of neural network models are undoubted, but at present, traditional neural networks can no longer meet the needs of society. As a result, a large number of neural network variants began to emerge in an effort to improve the performance of recognition models [1–3]. Liu et al. [3] built an attention-enhanced DenseNet neural network model by adding a hybrid attention mechanism on the basis of the neural network. The improved model can pay more attention to feature information, and the accuracy is significantly improved compared with other models. Sathya et al. [4] optimized the convolutional neural network and constructed the Reconstruction Disease Awareness Convolutional neural Network (RDA-CNN), which can convert low-resolution images into super-resolution images to achieve the purpose of improving the recognition accuracy of the model. Finally, the experimental results show that compared with the basic convolutional CNN, The performance of the new recognition model is improved by 4–6%. Wang et al. [5] introduced MobileNet structure and enhanced attention mechanism on the basis of neural network to construct the ADSNN-BO model, and used Bayesian optimization method to optimize the model parameters. Finally, they applied the optimized network to identify diseases. Bari et al. [6] introduced the RPN architecture in the basic line of the convolutional neural network, and constructed a new recognition model Faster R-CNN, and used the new model to identify the diseases. Through comparative experiments, it is also proved that the performance of the optimized model is better than the original model.

Using optimization mechanism to optimize the recognition model can indeed improve the performance of the recognition model, but the recognition accuracy of the model does not seem to be able to meet the expectations. Therefore, more optimization methods have been applied to the optimization of recognition models. In recent years, swarm intelligence algorithm [7–9] has become a research hotspot because of its excellent optimization performance, and has been widely used in the optimization of various problems, such as medical disease prediction [10–12], path optimization [13–15], hyperparameter optimization [16–18] and other fields. Based on this, a large number of experiments have tried to apply swarm intelligence algorithms to optimize the parameters and structure of the recognition model [19], and have achieved promising results. Lu et al. [20] used the improved whale algorithm to optimize the hyperparameters of the convolutional neural network, and applied the optimized model to the recognition of diseases. Finally, the experimental results show that the performance of the optimized model is better than the original model and several other common models. Zhu et al. integrated the particle swarm optimization algorithm into the classical convolutional neural network LeNet, and used the PSO algorithm to automatically select the hyperparameters of the model to construct a new PSO-LeNet [21] model. Comparative experiments show that the proposed model has commendable overall performance.

Although the integration of swarm intelligence algorithm into neural network is an effective way to optimize neural network, with the deepening of practical application, the disadvantages of swarm intelligence algorithm begin to appear. Based on the problems of low initial solution quality and imbalance between development and exploration, a large number of experiments have been carried out to optimize the swarm intelligence algorithm. In order to improve the performance of the whale optimization algorithm, Liu et al. introduced the Levy flight in the whale optimization algorithm, introduced a new convergent double adaptive weight, and also introduced a new mechanism for judging the predation state of whales. Experiments show that the performance of the proposed EGE-WOA [22] algorithm has been improved. Huang et al. proposed an improved particle swarm optimization algorithm with negative gradient perturbation and binary tree depth-first strategy, named GB-PSO [23], which has been experimentally proven to outperform some of the most advanced PSO algorithms in terms of search performance. The MDM-GWO [24] algorithm proposed by Singh et al. combines a new updated search mechanism, improved control parameters, mutation-driven scheme and greedy selection method in the search process of gray wolf optimization algorithm, and simulation experiments show that the MDM-GWO algorithm has superiority in multiple indicators. It can be seen that it is feasible to introduce optimization strategies into the swarm intelligence algorithm to improve the performance of the swarm intelligence algorithm.

In summary, the application of swarm intelligence algorithm to optimize the parameters of the recognition model can effectively improve the performance of the recognition model, and the optimization of the swarm intelligence algorithm can improve the optimization ability of the swarm intelligence algorithm. Therefore, this paper uses the optimized hunger search algorithm to optimize the parameters of the neural network in order to improve the performance of the model.

The structure of the article is as follows: the second part introduces the experimental data; The third part introduces the experimental methods; The fourth part introduces the experimental setup and analyzes the experimental results; Part V summarizes the research and presents future work.

## Data collection

**Fig 1:**
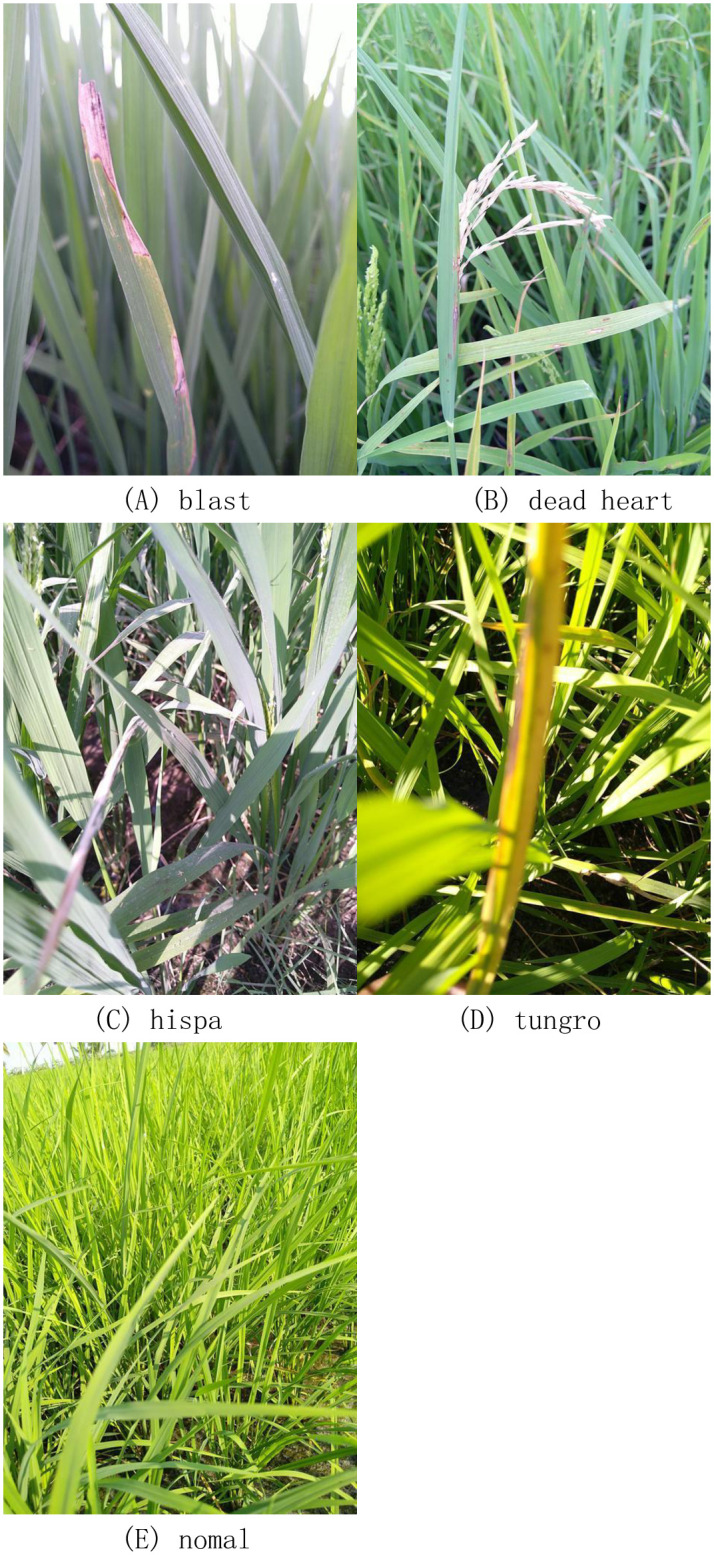
Dataset images. A.blast, B.dead heart, C.hispa, D.tungro, E.normal.

The experimental data came from the open source platform https://www.kaggle.com/datasets, and a total of 7626 rice leaf images were collected. All images were taken from the field, without complex background processing, compared with other datasets collected in the laboratory, this experimental dataset is more realistic, and more convenient for subsequent application in the field. The dataset includes four diseases, namely blast, dead heart, hispa, and tungro, of which 1738 blast disease images and dead. There were 1442 images of HEART disease, 1594 images of HISPA disease, 1088 images of Tugro disease, and 1764 images of normal. An example of a data image is shown in Fig 1.

## Methods

### Hunger Game search

**Fig 2:**
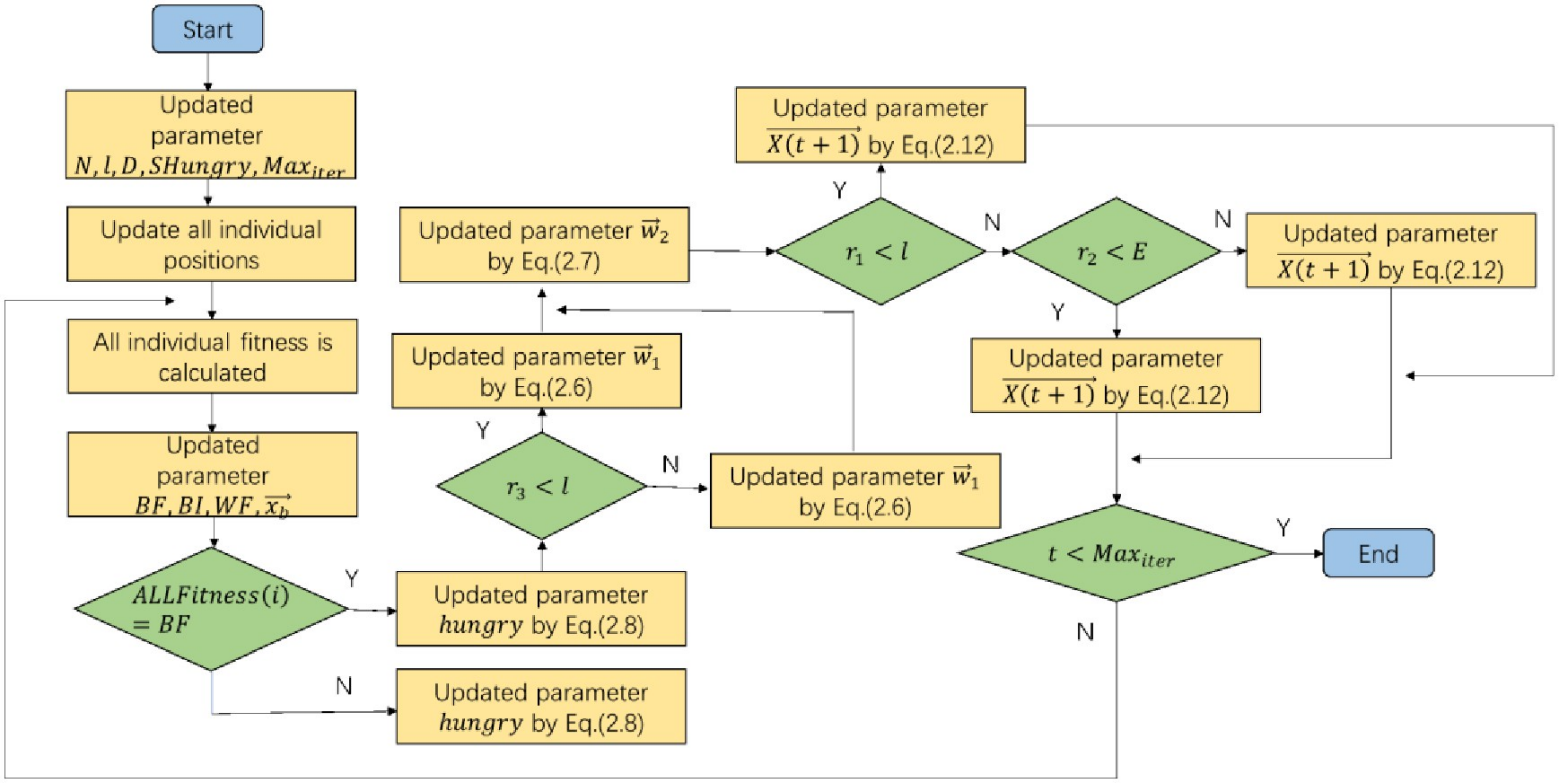
Flowchart of HGS.

The Hunger Games Search (HGS) algorithm is an original algorithm proposed by Yang et al. [25] in 2021. Its core concept is based on the thinking logic that animals in nature make decisions, search dynamically and take practical actions depending on the feeling of “hunger”, and the feeling of “hunger” is the key for animals to maintain their balance in nature. From the perspective of mathematical theory, hunger search is to construct the decision-making, search and action steps of animals in nature based on the sense of “hunger” by applying the adaptive weight design method. Different “hunger” weights represent different impacts of different “hunger” on animal decision-making, search and action. The flow chart of hunger game search algorithm is shown in Fig 2.

The Hunger Games search algorithm is mathematically modeled as follows:

$$\begin{array}{c} \vec{X(t+1)}=\{\begin{array}{c} \vec{X(t)}\left(1+\text{randn}\left(1\right)\right),r_{1}<l\\ \vec{W_{1}}\cdot \vec{X_{b}}+\vec{R}\cdot \vec{W_{2}}|\vec{X_{b}}-\vec{X(t)}|,r_{1}>l,r_{2}>E\\ \vec{W_{1}}\cdot \vec{X_{b}}-\vec{R}\cdot \vec{W_{2}}|\vec{X_{b}}-\vec{X(t)}|,r_{1}>l,r_{2}<E \end{array} \end{array}$$ (1)

Where *X*(*t*) denotes each individual location, *t* represents the current number of iterations of the overall algorithm, *randn*(1) is a random number that satisfies the standard normal distribution, *r*_1_,*r*_2_ is a random number between [0, 1]. The constant *l* is a constant set to improve the performance of the algorithm, and its value is set by experimental verification in the original paper.*W*_1_ and *W*_2_ are the starvation parameters, which are calculated below. *X*_*b*_ represents the position of the best individual in the current iteration, *R* is explained below.

In practice, the algorithm will set the upper and lower bounds. Therefore, to ensure that the search is within the specified range, the mathematical formula is set as follows:

$$\begin{array}{c} \vec{R}=\left(2*\;\text{rand}\;-1\right)*A \end{array}$$ (2)

The formula for calculating *A* is as follows:

$$\begin{array}{c} A=2*(1-\frac{t}{Max_{iter}}) \end{array}$$ (3)

The parameter *E* is calculated as follows:

$$\begin{array}{c} E=\text{sec}\;h(|F(i)-BF|) \end{array}$$ (4)

Where *i* ∈ (1, 2, ⋯, *s*) in the optimization process of the algorithm, the fitness of each individual will be calculated, *F*(*i*) represents the fitness value of each individual.*BF* is the best fitness in all iterations so far.

sec *h* is a hyperbolic function that is computed as follows:

$$\begin{array}{c} \text{sec}\;h\left(x\right)=\frac{2}{e^{x}+e^{-x}} \end{array}$$ (5)

HGS algorithm is constructed based on the principle of hunger-driven behavior activity, so two parameters representing hunger are constructed, which are *W*_1_ and *W*_2_, specifically representing the weight of hunger. The calculation formula of *W*_1_ and *W*_2_ are as follows:

$$\begin{array}{c} \vec{W_{1}\left(i\right)}=\{\begin{array}{c} \;\text{hungry}\;\left(i\right)\cdot \frac{N}{\;\text{SHungry}\;}*r_{4},r_{3}<l\\ 1,r_{3}>l \end{array} \end{array}$$ (6)

$$\begin{array}{c} \vec{W_{2}\left(i\right)}=\left(1-\text{exp}\;\left(-|\text{hungry}\left(i\right)-SHungry|\right)\right)*r_{5}*2 \end{array}$$ (7)

Where *hungry*(*i*) represents the hunger degree of each individual, *N* represents the number of populations, *SHungry* is the sum of the hunger degree of all individuals by adding the hunger degree of all individuals. *r*_3_, *r*_4_, *r*_5_ is a random number between [0, 1].

The formula for calculating *hungry*(*i*) is as follows:

$$\begin{array}{c} \;\text{hungry}\;\left(i\right)=\{\begin{array}{c} 0,\;\text{AllFitness}\;(i)=BF\\ \text{hung}\;y(i)+H,\;\text{else}\; \end{array} \end{array}$$ (8)

Where *AllFitness*(*i*) holds all the values of *F*(*i*). The formula for the calculation of *H* is as follows:

$$\begin{array}{c} H=\{\begin{array}{c} LH*(1+r),TH<LH\\ TH,\;\text{else}\; \end{array} \end{array}$$ (9)

The parameter *TH* is calculated as follows:

$$\begin{array}{c} TH=\frac{F(i)-BF}{WF-BF}*r_{6}*2*\left(UB-LB\right) \end{array}$$ (10)

Where *BF* is the current optimal fitness, *WF* is the current worst fitness, *r*_6_ is a random number between [0, 1]. *UB* and *LB* are the upper and lower limits of the search, respectively. *LH* is a lower bound on *H*, which is also a fixed parameter determined experimentally in the original paper.

HGS algorithmic pseudocode is as follows:

Algorithm 1 pseudo-code of HGS

Input: The population size *N*, the maximum number of iterations *T* and the variable dimension *D*.

Output: The optimal search agent *X*_*b*_ and its fitness value *BF*.

Initialize the parameters *N*, *T*, *l*, *D*, *Shungry*

Tent map initialize the positions of Individuals *X*_*i*_(*i* = 1, 2, ⋯*N*)

*r*_1_, *r*_2_, ⋯*r*_6_

While (*t* ≤ *T*)

 Calculate the fitness of all Individuals

 Update *BF*, *WF*, *X*_*b*_, *BI*

 Calculate the *Hungry* by Eq (8)

 Calculate the *overrightarrowW*_1_ by Eq (6)

 Calculate the *overrightarrowW*_2_ by Eq (7)

 For each Individuals

  Calculate *E* by Eq (4)

  Update *R* by Eq (2)

  Update positions by Eq (1)

 End for

 *t* = *t* + 1

End While

Although swarm intelligence algorithms have strong optimization ability, it is found that most swarm intelligence algorithms have the problem of imbalance between exploration and exploitation in the actual use of the process. In order to solve the imbalance problem between the exploration and development of the hunger game search algorithm, this experiment also solves the problem that the hunger game search algorithm population initialization uses a random way to generate, which makes the initial population distribution in the search space uneven and low ergodic, which leads to the algorithm’s incomplete search in the search space and affects the daily optimization performance and search efficiency of the algorithm. In this experiment, the adaptive weight mechanism and chaotic mapping mechanism are integrated on the basis of the Hunger Game search algorithm.

### Adaptive weight

Adaptive Weight is a dynamic adjustment algorithm that is commonly used to optimize algorithms and machine learning models to improve their performance. In this experiment, in order to solve the imbalance problem between exploration and exploitation in the hungry search algorithm, an adaptive weight mechanism is introduced into the location update.

The mathematical formula of the adaptive weight mechanism is as follows:

$$\begin{array}{c} w=\{\begin{array}{c} 1/2{\left[1+\text{cos}\;(\frac{\pi t}{{\text{Max}}_{\text{iter}}})\right]}^{\frac{1}{K}},t\leq \frac{{\text{Max}}_{\text{iter}}}{2}\\ 1/2{\left[1-\text{cos}\;(\pi +\frac{\pi t}{{\text{Max}}_{\text{iter}}})\right]}^{\frac{1}{K}},t>\frac{{\text{Max}}_{\text{iter}}}{2} \end{array} \end{array}$$ (11)

Where *K* represents the regulation coefficient and *t* represents the number of iterations. Max_iter_ stands for the maximum iteration.

The improved Hunger Games search algorithm position update formula is as follows:

$$\begin{array}{c} \vec{X(t+1)}=\{\begin{array}{c} \vec{W_{1}}\cdot \vec{X_{b}}+w\cdot \vec{R}\cdot \vec{W_{2}}|\vec{X_{b}}-\vec{X(t)}|,r_{1}>l,r_{2}>E\\ \vec{W_{1}}\cdot \vec{X_{b}}-w\cdot \vec{R}\cdot \vec{W_{2}}|\vec{X_{b}}-\vec{X(t)}|,r_{1}>l,r_{2}<E \end{array} \end{array}$$ (12)

The weight factor proposed in this paper decreases slowly at the beginning of generational selection, and the algorithm can maintain a good global exploration ability: the weight decreases rapidly after a certain number of iterations, so that the algorithm can search for the optimal solution more finely in the local development stage. The algorithm with adaptive weights added is named AHGS.

### Chaotic mapping

Chaotic Mapping refers to a class of nonlinear maps with chaotic behavior. Their properties are complex, including sensitive dependence on initial conditions, periodic orbits and randomness. Chaotic maps are widely used in physics, engineering, computer science, information theory and other fields.

A tent map, in mathematics, is a piecewise linear map, so named because its function image resembles a tent. In addition, it is also a two-dimensional chaotic map, which is widely used in chaotic encryption systems (such as image encryption), and is often used in the generation of mixed spread spectrum codes, the construction of chaotic encryption systems and the implementation of chaotic optimal selection algorithms.

The mathematical expression of Tent chaotic map is as follows:

$$\begin{array}{c} x_{t+1}=f(x_{t})=\{\begin{array}{c} \frac{x_{t}}{\alpha },x_{t}\in \left[0,\alpha \right]\\ \frac{1-x_{t}}{\left(1-\alpha \right)},x_{t}\in \left[\alpha ,1\right] \end{array} \end{array}$$ (13)

Where *alpha* is a random number with a value range of [0, 1]. Chaotic maps are widely used in information encryption, random number generation, dynamic system modeling and so on. Among them, the chaotic random number generator is to use some properties of chaotic maps, such as unpredictability and high chaos, to generate high-quality random number sequences.

In summary, chaotic maps are very useful mathematical tools that can be used to study the dynamics of physical systems, information encryption, random number generation and other problems.

In this experiment, the chaotic mapping mechanism is integrated into the population initialization of the Hunger Games search algorithm, which enhances the quality and distribution uniformity of the initial population, and helps the algorithm to carry out a more comprehensive search space. At the same time, the Hunger Games search algorithm sets six randomly generated random parameters with values in the range of [0, 1]. In this experiment, in order to improve the performance of the Hunger Games search algorithm, the chaotic mapping mechanism is also added to the generation of random parameter *r*_1_, *r*_2_, ⋯, *r*_6_.

### Googlenet

**Fig 3:**
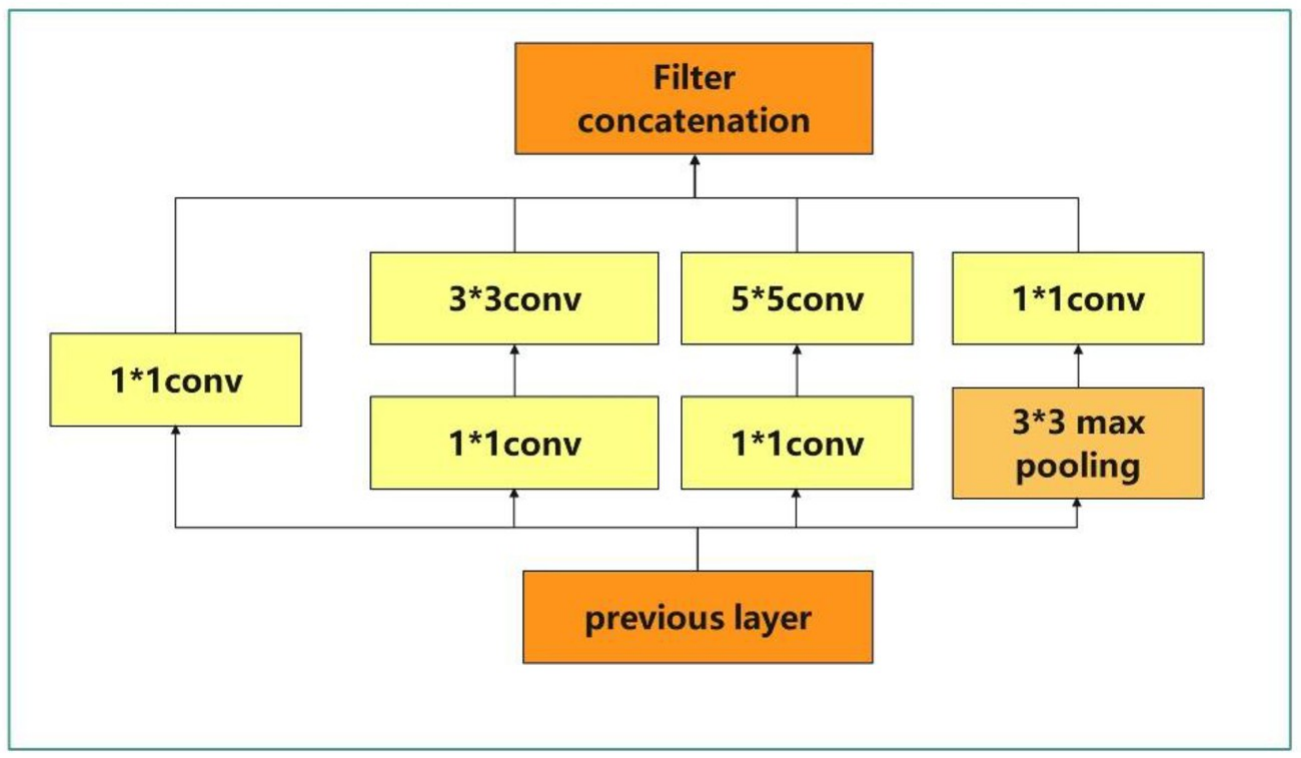
Inception block structure.

The basic convolution block in GoogLeNet is called Inception block, and Inception block is complex in structure, as shown in Fig 3 below. There are 4 parallel lines in the Inception block. The first three lines use convolution layers with window sizes of 1×1, 3×3 and 5×5 to extract information under different spatial sizes, and the middle two lines first do 1×1 convolution on the input to reduce the number of input channels and reduce the complexity of the model. The fourth line uses a 3×3 Max pooling layer followed by a 1×1 convolutional layer to vary the number of channels. All four lines use appropriate padding to match the input to the output height and width. Finally, the outputs of each line are concatenated in the channel dimension and transmitted backward.

### ATHGS-Googlenet

**Fig 4:**
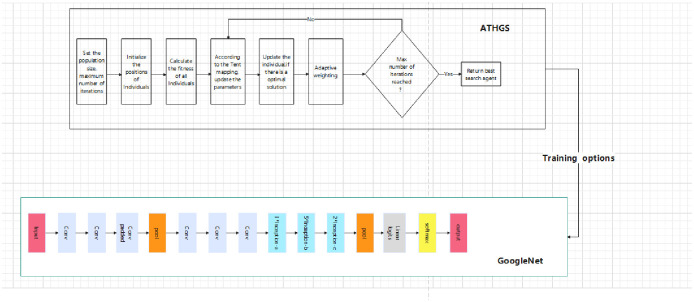
Flowchart of ATHGS-Googlenet.

The swarm intelligence algorithm with two improved algorithms is named ATHGS, where A is taken from the first letter of the adaptive weight and T is taken from the first letter of the Tent chaos map. In this experiment, the improved ATHGS algorithm is applied to adaptively find the optimal values of GoogLeNet learning rate, number of layers, and batch size, and a new model is constructed based on it, named Athgs-Googlenet, and the flow chart is shown in Fig 4.

Construct the fitness function with accuracy as follows:

$$\begin{array}{c} \;\text{Fitness}\;=\frac{\sum_{i=1}^{G}acc_{i}}{G} \end{array}$$ (14)

The pseudocode for the ATHGS-GoogLeNet model is as follows:

Algorithm 2 pseudo-code of ATHGS-GoogLeNet

Input:*N*, *D*, *T*, *K*_1_, *K*_2_, *K*_3_, *K*_4_,Hyperparameter EvalFunction

Output:Optimized deep learning parameters *w*&*b*

Initialize ATHGS algorithm population (including each individual’s position and deep learning parameters *w*&*b*, *a*, *A*, *C*)

Enter the training set(30% test set, 70% training set, the dataset is random)

For(*t* ≤ *T*)

 For(*i* ≤ *N*)

  Calculate the fitness

  Update the best individual location

 End For

 Update algorithm parameters

 (*K*_1_, *K*_2_, *K*_3_, *K*_4_) = Hyperparameter EvalFunction(current optimal position)

End For

Stochastic Gradient Descent with momentum(SGDM)

Update *w*&*b* using SGDM with hyperparameters *K*_1_, *K*_2_, *K*_3_, *K*_4_ Return *w*&*b*

## Experimental setup and results

### Experimental setup

The experiments were all carried out in the laboratory, the recognition system was implemented by MATLAB, all experiments were repeated 30 times independently, and the parameter *l* in the algorithm was 0.08 according to the original text. Firstly, in order to verify the effectiveness of the proposed algorithm ATHGS, the algorithm proposed in this experiment is applied to three engineering experimental design problems with AHGS, THGS, original HGS algorithm, WOA, GWO and PSO, namely pressure vessel design problem, three-rod truss design problem and tension spring design problem. Secondly, in order to further verify the effectiveness of the proposed algorithm, the ATHGS algorithm is compared with the WOA, GWO and PSO algorithms for the CEC 2017 benchmark function, and the results are displayed in the form of convergence curves and the values of the optimal solution. Finally, in order to verify the effectiveness of the recognition model ATHGS-GoogleNet developed in this experiment, seven basic neural network models were selected and compared with the GoogleNet model improved by the above six swarm intelligence algorithms, and the seven basic neural network models were AlexNet, DenseNet 201, GoogleNet, Inception V3, ResNet 18, ResNet 50, VGG16. In order to verify the performance of the identification model in this study, three common performance indicators were selected as evaluation indicators, namely accuracy [26], sensitivity [27] and precision [28].

### Improved algorithm comparison results

#### Pressure vessel design issues

Pressure vessel design refers to the design and calculation of vessels under pressure to ensure their safety and reliability under working conditions. Common pressure vessels include gas storage tanks, oil tanks and chemical equipment. When designing a pressure vessel, it is necessary to consider factors such as material strength, thickness, size, structural form, seam welding and other factors of the vessel to meet the required working pressure and safety requirements. Table 1 shows the optimization results of pressure vessel design problems, where TS represents the thickness of the vessel wall, TH represents the thickness of the hemisphere head, R represents the inner radius, and L represents the length of the cylindrical section.

**Table 1:** Optimization results of pressure vessel design issues.

Name	Fitness value	TS	TH	R	L
ATHGS	8255.4	1.3	0.6	67.4	44.8
AHGS	8507.0	1.3	0.7	67.4	10.0
THGS	8528.7	1.3	0.6	67.4	10.0
HGS	9682.2	1.3	0.7	67.4	10.0
WOA	10090.1	1.3	0.6	67.4	10.0
GWO	11163.7	1.3	0.6	67.4	10.0
PSO	11694.4	1.3	0.6	67.4	10.0

The smaller the fitness value, the better the optimization performance of the algorithm. As can be seen from Table 1, the first algorithm proposed in this paper, ATHGS, has a fitness value of 8255.4, which is the smallest among all algorithms. This was followed by AHGS with a fitness value of 8507.0, followed by THGS with a fitness value of 8528.7 and then HGS with a fitness value of 9682.2. WOA, GWO and PSO ranked last, with an adaptability of more than 10,000. From the experimental results, it can be seen that the ATHGS algorithm proposed in this paper has the best optimization performance in the pressure vessel design problem.

#### Three-pole truss design issues

A three-pole truss is a structure consisting of three members that is often used in engineering to support and bear loads. The design of the three-pole truss involves the material strength of the member, the cross-sectional size of the member, the way the node is connected, etc. Through reasonable design, it can be ensured that the truss structure has sufficient rigidity and bearing capacity to meet the design requirements. Table 2 shows the optimization results of the three-pole truss design problem, where X1 and X2 represent different cross-sectional areas.

**Table 2:** Optimization results of the three-pole truss design issues.

Name	Fitness value	X1	X2
ATHGS	263.9	0.8	0.4
THGS	264.0	0.8	0.4
HGS	264.0	0.8	0.4
WOA	264.1	0.8	0.5
GWO	264.1	0.8	0.4
PSO	264.1	0.8	0.4

As in Table 1, the smaller the fitness value, the better the optimization performance of the algorithm. It can be seen from Table 2 that in the three-pole truss design problem, the fitness values of all algorithms are relatively similar, which means that all algorithms have similar optimization performance in the three-pole truss design problem, but even so, the algorithm ATHGS proposed in this paper still has the best optimization performance, and the fitness value is 263.9. Although AHGS and ATHGS have the same fitness values in three-pole truss design problems, ATHGS outperforms AHGS in other engineering design problems.

#### Tension spring design issues

A tension spring is a spring with elastic deformation characteristics that provides stability and equilibrium for mechanical systems by applying tension. The design of tension springs takes into account factors such as material characteristics, size, and stiffness coefficient of the spring. Reasonable design can ensure that the tension spring has the appropriate tension during the working process to meet the requirements of the system. Table 1 shows the optimization results of tension spring design problems. where d represents the diameter of the spring coil, D represents the diameter of the spring coil, and P represents the number of coils.

Same as Tables 1 and 2, the smaller the fitness value, the better the optimization performance of the algorithm. Since the fitness values of all algorithms are less than 1 in the tension spring design problem, in order to clearly show the gap between the fitness values of each algorithm, the results of this experiment retain 5 decimal places after the decimal point. As can be seen from Table 3, only the fitness value of the algorithm proposed in this paper is less than 0.013, and the fitness value of the other algorithms is greater than 0.013. The fitness values of PSO reached above 0.016. It is not difficult to see that the algorithm ATHGS proposed in this paper has the best optimization performance in the design problem of tension spring.

**Table 3:** Optimization results of tension spring design issues.

Name	Fitness value	d	D	p
ATHGS	0.01272	0.05342	0.39990	9.14350
AHGS	0.01312	0.05291	0.38673	9.75120
THGS	0.01314	0.05554	0.45638	7.19110
HGS	0.01343	0.05967	0.58071	4.65030
WOA	0.01349	0.05864	0.54827	5.15160
GWO	0.01363	0.05503	0.44225	7.65790
PSO	0.01650	0.05930	0.56897	4.82660

#### The results of functional tests

In order to further verify the effectiveness of the proposed algorithm, the proposed algorithm is compared with the WOA, GWO and PSO algorithms for CEC 2017 benchmark functions. Table 4 shows the results of the mean level indicators.

**Table 4:** Compare the test functions.

Name/Algorithm	ATHGS	WOA	GWO	PSO
F1	8.14E-02	7.56E-10	7.56E-10	1.42E-08
F2	8.81E-01	7.56E-10	3.17E-09	3.73E-07
F3	4.96E-01	6.58E-03	1.44E-02	8.02E-06
F4	4.09E-01	7.56E-10	7.91E-07	7.56E-10
F5	4.73E-02	7.56E-10	8.31E-07	1.56E-09
F6	2.48E-03	7.56E-10	1.16E-02	7.56E-10
F7	3.52E-01	8.53E-10	5.87E-07	6.83E-05
F8	9.19E-01	7.56E-10	7.95E-04	1.56E-09
F9	5.66E-01	8.03E-10	9.17E-07	2.66E-09
F10	5.15E-01	7.56E-10	3.02E-02	7.56E-10
F11	1.31E-01	7.56E-10	7.56E-10	1.36E-02
F12	2.18E-01	7.56E-10	8.53E-10	1.20E-08
F13	3.13E-01	7.56E-10	7.56E-10	7.56E-10
F14	2.73E-01	1.01E-06	7.41E-04	8.49E-02
F15	3.62E-01	7.56E-10	8.50E-09	7.56E-10
F16	3.98E-01	1.09E-09	8.36E-05	2.22E-04
F17	7.98E-01	1.66E-09	5.58E-04	3.48E-04
F18	7.54E-01	7.56E-10	5.06E-02	3.42E-01
F19	3.88E-01	7.56E-10	1.56E-09	7.56E-10
F20	9.73E-01	2.62E-08	2.04E-03	7.12E-05
F21	7.17E-01	7.56E-10	7.56E-10	6.82E-01
F22	8.66E-02	7.56E-10	9.14E-08	9.00E-09
F23	1.02E-01	7.56E-10	1.47E-09	7.56E-10
F24	1.00E+00	4.34E-04	7.56E-10	7.56E-10
F25	1.00E+00	1.25E-01	7.56E-10	7.56E-10
F26	1.00E+00	4.19E-04	7.56E-10	7.56E-10
F27	1.00E+00	7.56E-10	7.56E-10	7.56E-10
F28	1.00E+00	1.95E-04	7.56E-10	7.56E-10
F29	5.00E-01	1.11E-09	8.03E-10	7.56E-10
F30	4.08E-01	2.56E-09	7.56E-10	7.56E-10
mean level	2.068333333	4.468666667	2.937333333	3.444
rank	1	4	2	3

As can be seen from Table 4, the optimal solution of the test function obtained by ATHGS is better than that of other algorithms, and the mean level value is also the smallest, ranking first overall.

**Fig 5:**
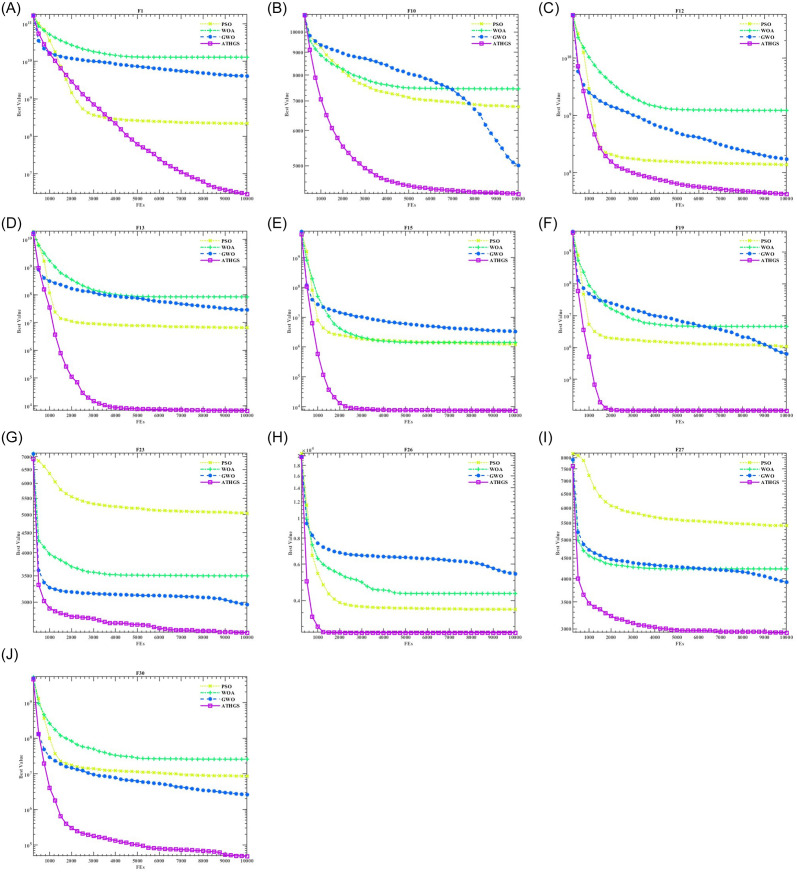
Algorithm convergence diagram.

The results of the convergence curves of ATHGS with WOA, GWO and PSO are shown in Fig 5. Select the 10 best functions to display.

As can be seen from the Fig 5, the ATHGS algorithm tends to converge in the early stage, which is better than other algorithms, and at the same time, it also shows its own advantages in optimization accuracy and convergence speed.

### Identify model comparison results

#### Experimental results of the model presented in this paper

**Fig 6:**
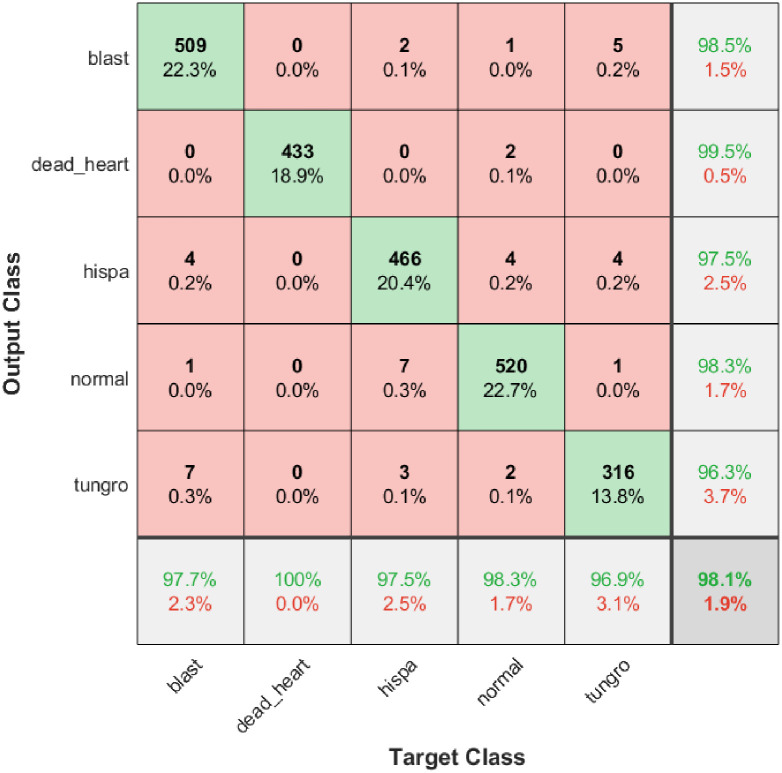
ATHGS-Googlenet confusion matrix.

The confusion matrix results of the ATHGS-GoogleNet model proposed in this experiment are shown in Fig 6, in which each row of the first five rows represents the recognition category of the experimental sample, the green font in each cell in the last row represents the recognition sensitivity of each category, each column in the first five columns represents the real category of the experimental sample, the green font in each cell of the last column represents the recognition accuracy of each category, and the green font in the intersection cell of the last row and the last column represents the model recognition accuracy. As can be seen from Fig 6, the sensitivity of the ATHGS-GoogleNet model for all diseases can reach the lowest 96.9%, the sensitivity of identifying disease 2 reaches 100%, the identification precision of all diseases can reach the lowest 96.3%, the highest can reach 99.5%, and the overall recognition accuracy of the model reaches 98.1%. It can be seen that all indicators of the ATHGS-GoogleNet model have more than 95% results.

**Fig 7:**
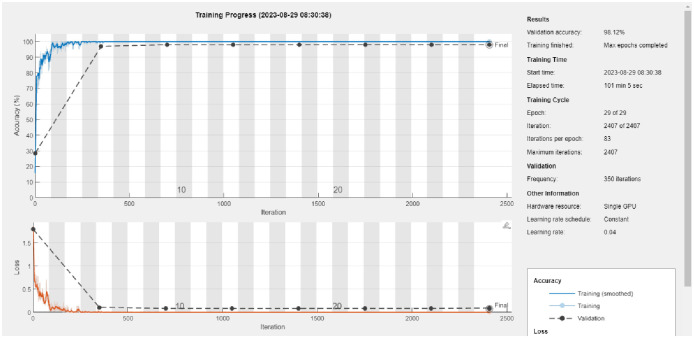
ATHGS-Googlenet model training progress.

The ATHGS-GoogleNet model training progress graph is shown in Fig 7, where the blue line represents the accuracy rate, the red line represents the loss rate, and the right side represents the setpoint of each parameter. It can be seen from the two curves that the accuracy rate of ATHGS-GoogleNet is obviously increasing as the training progress progresses, and the loss rate is decreasing until it stabilizes.

#### Basic neural network model experimental results

**Fig 8:**
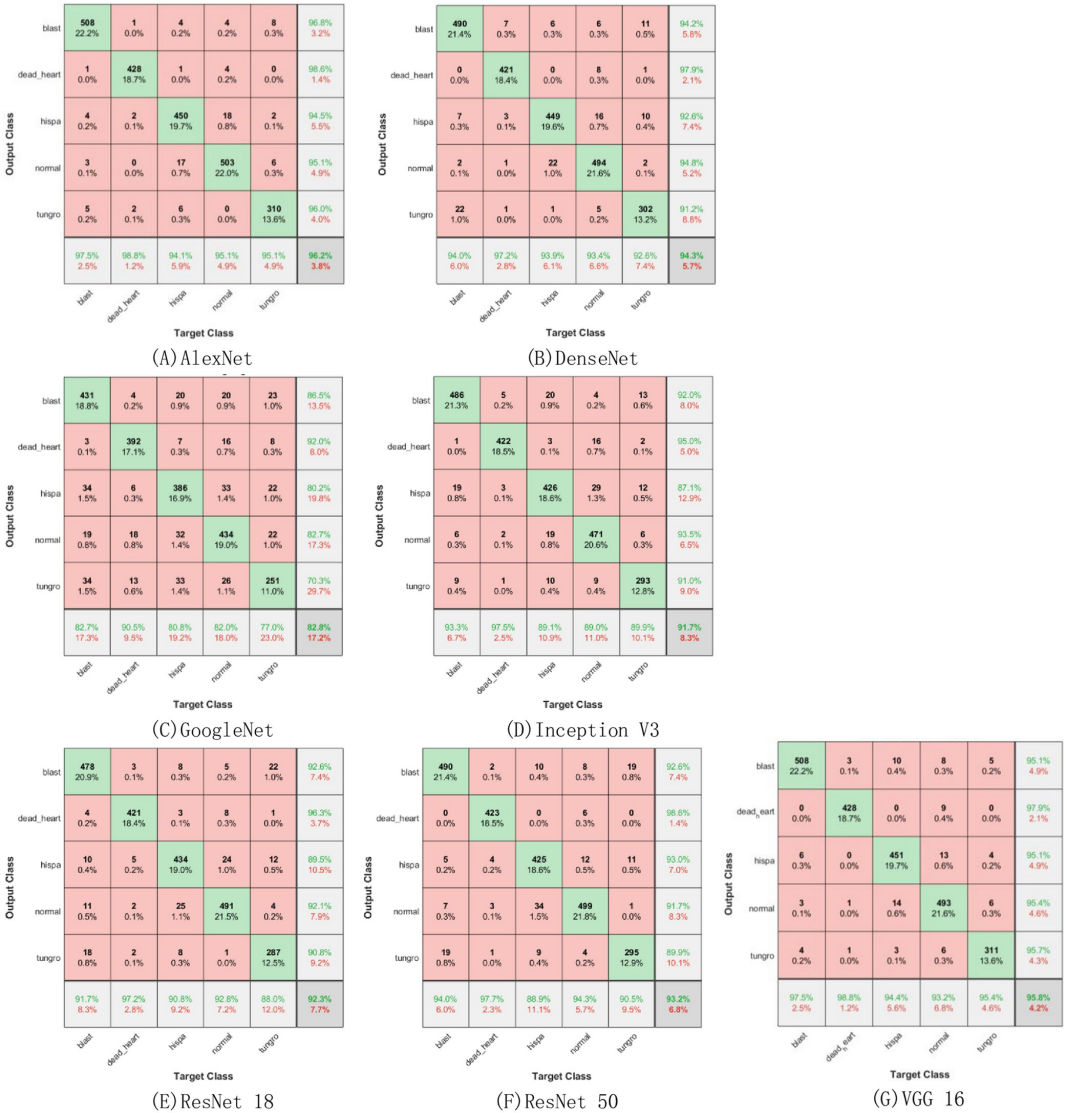
Basic neural network model confusion matrix. A.Alexnet, B.Densenet, C.Googlenet, D.Inceptionv3, E.Resnet 18, F.Resnet 50, G.VGG16.

The results of the confusion matrix of the seven basic neural network models are shown in Fig 8, and the interpretation of Fig 8 is the same as Fig 6. As can be seen from Fig 8, AlexNet has the highest accuracy rate, reaching 96.2%, and GoogleNet has the lowest accuracy rate, only 82.8%. The accuracy of ATHGS-GoogleNet is 1.9 percentage points higher than that of AlexNet, and 15.8 percentage points higher than that of GoogleNet, and the accuracy of ATHGS-GoogleNet is improved to varying degrees compared with other basic neural network models, which can clearly see the effect of improvement on improving the accuracy of the model. For the evaluation index of sensitivity, the sensitivity of basic neural network models for the identification of a disease generally reaches more than 90%, of which AlexNet and VGG 16 have the highest recognition sensitivity to dead heart, reaching 98.8%, but there are also individual basic neural network models that show poor recognition sensitivity, and GoogleNet has the lowest recognition sensitivity to tungro, only 77.0%. Compared with all basic neural network models, the recognition sensitivity of ATHGS-GoogleNet has been improved to varying degrees, and compared with the basic GoogleNet, the recognition sensitivity for tungro has increased by 19.9 percentage points, and the improvement effect is very obvious. For the evaluation index of recognition precision, in the basic neural network model, GoogleNet provides the worst recognition precision, the recognition precision for tungro is only 70.3%, AlexNet and ResNet 50 provide the best recognition precision, of which AlexNet and ResNet for dead heart recognition precision of 98.6%. Compared with all basic neural network models, ATHGS-GoogleNet has improved precision to varying degrees, compared with the highest recognition precision, ATHGS-GoogleNet’s recognition precision for dead heart has reached 99.5%, an increase of 0.9 percentage points, and compared with the worst recognition precision, ATHGS-GoogleNet’s recognition precision for tungro has reached 96.3%, an increase of 26 percentage points. It can be seen that the improved method is of great help to improve the recognition sensitivity and precision of the model.

#### Experimental results of other swarm intelligence algorithms after optimization

**Fig 9:**
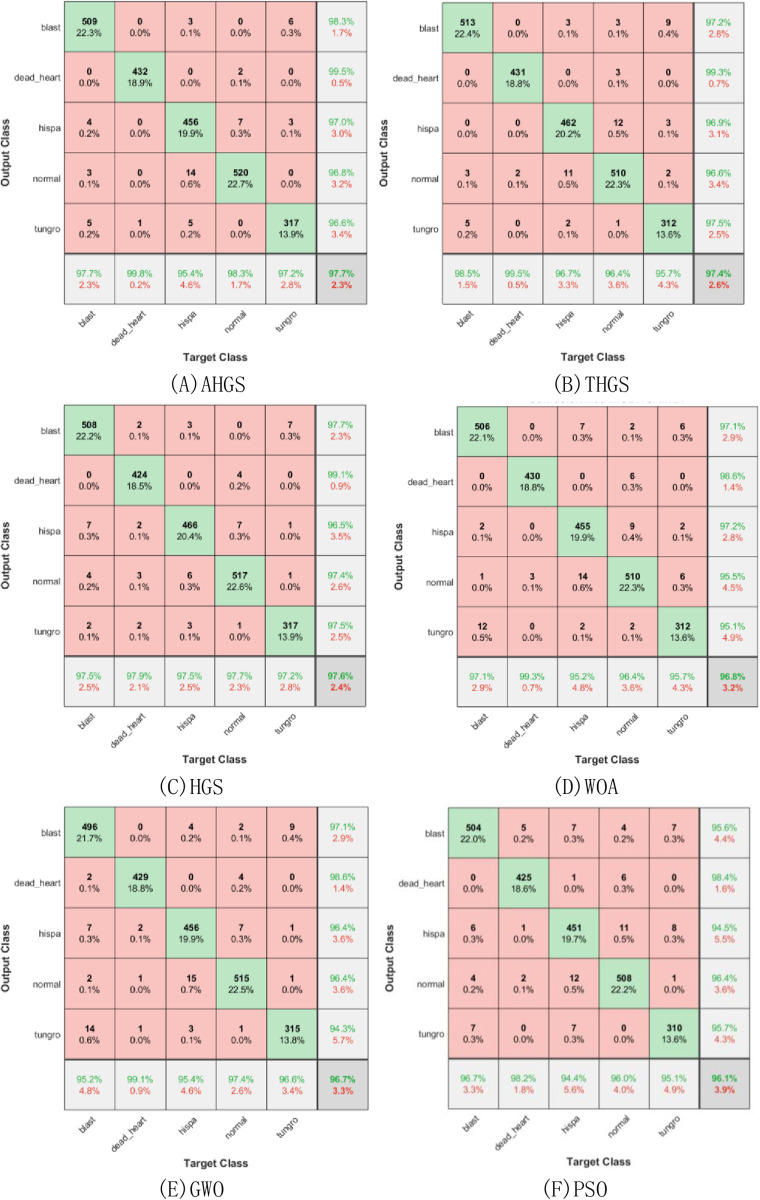
The crowd intelligence algorithm optimizes the confusion matrix of Googlenet. A.AHGS-Googlenet, B.THGS-Googlenet, C.HGS-Googlenet, D.WOA-Googlenet, E.GWO-Googlenet, F.PSO-Googlenet.

The confusion matrix result of the optimization of GoogLeNet by the swarm intelligence algorithm is shown in Fig 9, and Fig 9 is explained in the same way as Figs 6 and 8. As can be seen from Fig 9, AHGS-GoogleNet has the highest accuracy rate, reaching 97.7%, followed by THGS-GoogleNet, which is only 0.1 percentage points lower than AHGS-GoogleNet, reaching 97.6%, and the third is HGS-GoogleNet, with an accuracy rate of 97.4%, and WOA-GoogleNet and GWO-GoogleNet have similar accuracy in the fourth and fifthThe accuracy rate reached 96.8% and 96.7%, respectively, and PSO-GoogleNet had the lowest accuracy rate, only 96.1%. Compared with the ATHGS-GoogleNet proposed in this paper, ATHGS-GoogleNet still has the highest accuracy rate, compared with the original HGS-optimized HGS-GoogleNet. The improvement is 0.7 percentage points, compared with the lowest PSO-GoogleNet, there is a significant improvement, an increase of 2 percentage points, compared with other swarm intelligence algorithm optimization models, there are different degrees of improvement, it can be seen that the model optimized by the algorithm proposed in this paper has the highest accuracy. For the evaluation index of sensitivity, AHGS-GoogleNet, HGS-GoogleNet, WOA-GoogleNet and GWO have a recognition sensitivity of more than 99%, of which AHGS-GoogleNet provides the highest recognition sensitivity, AHGS-GoogleNet recognizes dead heart, the recognition sensitivity reaches 99.8%, followed by HGS-GoogleNet. Its sensitivity reached 99.5% when identifying dead heart, and WOA-GoogleNet and GWO-GoogleNet ranked third and fourth, respectively, and the recognition sensitivity reached 99.3% and 99.1% when identifying dead heart, respectively. PSO-GoogleNet ranked fifth, with a recognition sensitivity of 98.2% when identifying dead heart, but it also provided the lowest recognition sensitivity, and its identification sensitivity was only 94.4% when identifying HISPA diseases. However, compared with the ATHGS-GoogleNet proposed in this paper, ATHGS-GoogleNet still has the highest sensitivity, which is 0.2 percentage points higher than AHGS-GoogleNet, which ranks first among 6 other models improved by group intelligence algorithms, reaching 100%, which is a significant improvement compared with other models. For the evaluation index of precision, the highest precision of the six models in Fig 8 is more than 98%, of which AHGS-GoogleNet provides the highest precision, which reaches 99.5% precision when identifying dead heart, GWO-GoogleNet and PSO-GoogleNet provide poor precision, PSO-GoogleNet recognizes only 94.5% when identifying hispa. Compared with the precision of GWO-GoogleNet when identifying tungro, the ATHGS-GoogleNet proposed in this paper provides the best recognition precision, which reaches 99.5% in identifying dead hear, which has the same precision as the AHGS-GoogleNet, which ranks first in Fig 8, but when identifying other diseases. The recognition precision of ATHGS-GoogleNet is higher than that of AHGS-GoogleNet, and the precision of AHGS-GoogleNet is significantly higher than that of other models. It can be seen that the improved method proposed in this paper can effectively improve the recognition accuracy of the model.

## Discussion

In this study, a new swarm intelligence algorithm ATHGS and a new recognition model ATHGS-GoogleNet are proposed, which apply ATHGS to three engineering experimental designs and apply the model to rice disease identification. First, 7626 field rice disease images were collected as the dataset of this experiment, and the data sets were not preprocessed, which could ensure that the methods studied in this experiment could be more suitable for real field operations. Second, in order to improve the optimization performance of the original HGS algorithm, the adaptive weight mechanism and chaotic mapping mechanism are applied to optimize the algorithm, and a new algorithm ATHGS is constructed, in order to verify the effectiveness of the algorithm ATHGS proposed in this paper, ATHGS is applied to three engineering experimental design problems, and compared with other 6 swarm intelligent algorithms, it can be seen that the performance of the ATHGS algorithm proposed in this paper is indeed better than the other 6 algorithms. Thirdly, the optimized algorithm is applied to optimize the parameters of the GoogleNet network to improve the recognition performance of the basic network, and the newly established model is applied to identify rice diseases based on the collected dataset. Fourth, in order to verify the performance of the proposed model, six other group algorithms were applied to optimize the GoogleNet network, and seven basic common neural network models were selected to identify based on the experimental dataset, and compared with the model proposed in this paper for three evaluation indicators. Based on the experimental results, the model proposed in this experiment can be applied to the identification of rice diseases, which is helpful to reduce the occurrence of rice yield reduction due to diseases. However, the model proposed in this experimental experiment also has certain limitations, first of all, the dataset is too single, and the experimental dataset only comes from an open source platform; Second, more rice diseases were not included. In our future work, we plan to increase the number of datasets, enrich the types and sources of disease in the datasets, and find better algorithms for model optimization and improve model performance.
